# Subject-specific multivariate modeling for regenerative rehabilitation of bone healing

**DOI:** 10.1063/5.0273944

**Published:** 2025-10-24

**Authors:** Kylie E. Williams, Farhan Muhib, Ethan Dinh, Kelly E. Leguineche, Auveen Hajarizadeh, J. Walker Rosenthal, Tyler Guyer, Theo Seah, Nick J. Willett, Jeffrey A. Weiss, Robert E. Guldberg

**Affiliations:** 1 Phil and Penny Knight Campus for Accelerating Scientific Impact, Department of Bioengineering, University of Oregon, 1505 Franklin Blvd., Eugene, Oregon 97403, USA; 2 Department of Biomedical Engineering, University of Utah, 36 S Wasatch Dr., Salt Lake City, Utah 841123, USA; 3 Scientific Computing and Imaging Institute, University of Utah, 72 S Central Campus Dr., Salt Lake City, Utah 84112, USA; 4 Department of Orthopaedics, University of Utah, 201 Presidents' Cir., Salt Lake City, Utah 841123, USA

## Abstract

Rehabilitation can help promote functional restoration following surgical reconstruction of severe bone injuries, yet prescribed regimens are often conservative due to limited understanding of their impact on healing. This study examined rodent physical activity parameters, including wheel running duration, distance, bout frequency, bout duration, velocity, and rest time, assessing their combined impact on bone healing in 2 and 3 mm segmental defects. Artifical intelligence (AI)-based genetic programming generated high-accuracy nonlinear models, revealing a “goldilocks” phenomenon: some rehabilitation stimulates bone formation, but excessive activity is detrimental. Subject-specific finite element modeling showed that local defect compressive strains decreased only in injuries that achieved union and that the correlation between strain and healing evolved over time. These findings highlight the dynamic healing process, necessitating a subject-specific approach. While bone healing is often thought to be primarily driven by achieving optimal strain magnitudes, our results suggest a more complex reality. Bone healing depends not only on optimal strain magnitude but also on balancing activity and rest, which shifts with injury severity and healing progression. Overall, effective rehabilitation must consider injury stabilization, severity, and healing status while ensuring adequate rest to promote optimal bone regeneration.

## INTRODUCTION

Complex bone injuries continue to exhibit high complication rates and poor functional recovery, partially due to the insufficient understanding of how rehabilitative loading impacts bone healing. Severe bone defects require surgery to place stabilization hardware, followed by postoperative rehabilitation.^1^ Prescribed rehabilitation heavily relies on subjective patient feedback on pain levels, hard-to-interpret radiographs, and the intuition and experience of physicians.^2,3^ The lack of quantitative metrics to inform rehabilitation decisions has led to conservative recommendations that often involve weeks of non-loading.^4^ Although animal segmental bone defect models have shown that moderate loading starting 1 week after injury improved bone healing, these insights have had limited impact on clinical rehabilitation approaches.^5–7^ Therefore, a better understanding of how early rehabilitation conditions impact bones' endogenous potential to regenerate is crucial to aid in the clinical approach to post-injury rehabilitation. Furthermore, technology that enables quantitative subject-specific monitoring to inform clinical rehabilitation decisions is critical to advancing rehabilitation strategies.

Decades of literature have established that bone is a mechanoresponsive tissue.^5,6,8–15^ The idea that mechanical stimuli result in bone adaptation was advanced by Wolff, whose early theories promoted the idea that intact bones remodel in response to functional loading patterns.^16^ Since then, extensive research has found that loading can also aid in bone repair by improving callus formation, consolidation, and remodeling.^5,17–19^ However, research has also found that excessive loading can severely disrupt important regeneration processes like revascularization and callus consolidation, resulting in nonunion.^7,9^ Most loading strategies designed to facilitate bone healing have focused on structured loading regimens that are controlled by fixation plate stiffness or loading apparatuses, which have limited clinical applicability.^5,6,9,10,12,20–29^ Perren, Claes, and several others identified interfragmentary strain as a critical factor in fracture healing, which motivated a large focus on the local mechanical environment after bone injury.^30–33^

Across several animal and human studies, results have indicated that if fixation is either too flexible or too rigid, the healing might fail.^9,34–37^ For instance, if strains of high magnitude exist at a fracture site due to inadequate stability or excessive loading, fibrous tissue will remain, and the stabilizing callus will be unable to form. If such conditions continue for long enough, a fibrous nonunion will result.^34,35^ Paradoxically, some studies have also demonstrated that excessively rigid fixation may impair fracture healing as well.^5,17,38,39^ These studies often investigate one or two variables at a time (e.g., loading magnitude, loading rate, fixation plate stiffness, etc.). However, clinical rehabilitation following musculoskeletal injuries inevitably involves multiple rehabilitation parameters that simultaneously influence healing. Therefore, we used our segmental bone defect, ad lib wheel running, and revolution counters to track subject-specific activity and calculate six exercise parameters (running duration, running distance, number of running bouts, running bout duration, running velocity, and daily rest time) to ultimately investigate their multifactorial influence on bone healing.

Historically, load magnitude and timing are often viewed as the dominant factors influencing bone formation. For example, in a rat femoral defect model, Boerckel *et al.* sought to investigate how the timing of load onset impacted bone healing. They used a stabilization plate with a locking mechanism that changed plate stiffness when deployed. Immediately unlocking the plate postoperatively resulted in high magnitude loading that significantly inhibited the bone formation of large 8 mm defects treated with low dose bone morphogenetic protein-2 (BMP-2), primarily by disrupting revascularization. In contrast, unlocking the plate after callus formation (week 4) was beneficial to healing.^9^ Klosterhoff *et al.* also used a rat femoral defect model to investigate how low- and high-stiffness fixation hardware would impact bone healing but without dynamic changes in plate stiffness. They found that fixation with lower stiffness facilitated an increase in compressive strains within the regenerative niche during treadmill walking, which ultimately led to improved bridging rates and bone formation in a 6 mm defect treated with low-dose BMP-2.^5^ In this study, the lower stiffness hardware was relatively more rigid than the unlocked fixation strategy from Boerckel *et al.*, which inhibited healing, underscoring the need to characterize local strains more thoroughly.^5,9^ Throughout these studies, researchers utilized a segmental defect rodent model with a similar femur fixation hardware and surgical approaches as our current study. Importantly, the previous studies delivered local BMP-2, a well-established biologic that robustly induces ossification.^40–42^ The use of BMP-2 stimulation may complicate an understanding of the inherent mechanosensitive response by inducing earlier, more significant cartilage and mineral deposition.^43–45^

To eliminate the use of biologics, Williams *et al.* explored rehabilitation intensity as a means to increase loading magnitudes and improve functional bone healing after segmental bone injuries that were not treated with BMP-2.^46^ A segmental 2 mm bone defect in rat femurs exhibits variable spontaneous bridging rates, making it ideal for assessing the potential of rehabilitation to direct bone healing without the need for biologics. This also addresses a significant blind spot in the literature, which generally focuses on very large supercritical defects treated with biologics or small osteotomies that heal via intramembranous ossification. Animals exercised on a running wheel with or without friction applied, producing high (resistance) vs low (no resistance) intensity rehabilitation conditions. Resistance rehabilitation that began 1 week after injury resulted in increased compressive and shear strains within the regenerative niche, increased bone formation compared to sedentary counterparts, and restored femoral mechanical properties to intact strength.^46^ These previous studies have revealed the importance of loading onset and magnitude on both critically (6–8 mm) and near-critically sized bone defects (2 mm). Despite these crucial insights into the mechanobiology principles of bone healing, there remains a need for more studies to track and analyze multiple rehabilitation parameters to understand their combined influence on bone healing.

In this study, we sought to examine the combined effect of several rehabilitation parameters on bone injuries of two different sizes, motivated by the goal of subject-specific rehabilitation. We surgically created 2 and 3 mm bilateral segmental bone defects in rodent femurs that were stabilized with internal fixation plates. After surgery, we allowed animals to recover in sedentary conditions for one week and randomly assigned them to either sedentary conditions or one of three rehabilitation conditions. Experimental groups included sedentary controls, low distance rehabilitation controls (2 h/day of wheel access with limited distance), restricted rehabilitation (2 h/day of wheel access), and unrestricted rehabilitation (24/7 of wheel access). Rehabilitation parameters (running duration, running distance, number of running bouts, running bout duration, running velocity, and daily rest time) and subsequent bone healing were longitudinally monitored via revolution counters on the running wheels and radiographs and microCT scans, respectively. Explanted femora underwent histology to investigate how rehabilitation conditions impacted tissue-level adaptations for representative subjects. Subject-specific finite element (FE) analyses were also performed to track strains within the regenerative niche in response to rehabilitation conditions, mineralization status, and healing status. AI-based genetic programming algorithms were also used to evaluate the multifactorial effect of rehabilitation on bone healing outcomes. Building on the working theory that bone healing requires a balanced loading regimen, we hypothesized that restricted rehabilitation would increase the bridging rates and end point bone volume compared to unrestricted rehabilitation and sedentary controls. We also hypothesized that the impact of rehabilitation would depend on injury severity due to different biological needs and mechanosensitivity.

## RESULTS

**FIG. 1.**
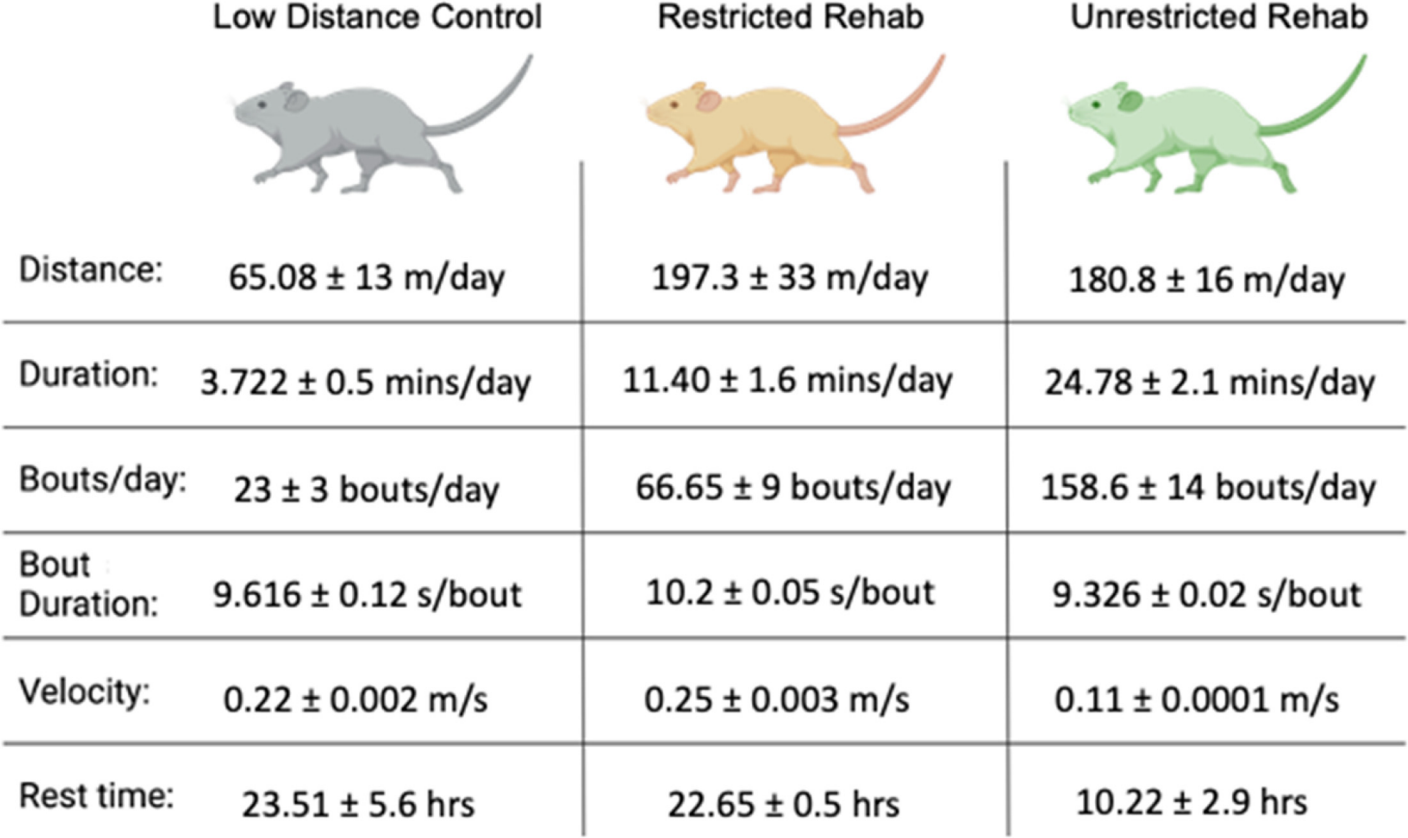
Rehabilitation conditions impacted several exercise parameters. See supplementary material Fig. 1 for longitudinal statistics on specific rehabilitation parameters. n = 6 for low distance control; n = 7 for restricted rehab, and n = 5 for unrestricted rehab. Graphic created with biorender.com. Mean ± SD.

Within this study, our experimental groups consisted of (1) sedentary controls; (2) low distance controls where rodents were housed with running wheels that were only unlocked, allowing rodents to run on the wheel from 8 to 10 pm each day; (3) restricted rehabilitation where rodents were housed in standard conditions and brought to the wheel cage for ad lib exercise from 8 to 10 pm each day; and (4) unrestricted rehabilitation where rodents were housed with running wheels that remained unlocked allowing ad lib 24/7 running access. Across the rehabilitation groups, we examined rodents' wheel running duration, running distance, number of running bouts, running bout duration, running velocity, and daily rest time (Fig. 1 and supplementary material Fig. 1). Longitudinal activity data revealed that hours of wheel access and housing conditions resulted in three distinct rehabilitation profiles (Fig. 1). Unexpectedly, both the restricted rehabilitation and unrestricted rehabilitation groups ran roughly the same daily distance although they were given 2 and 24 h of daily wheel access, respectively (Fig. 1). The low distance control group ran significantly less daily running distance, duration, and bouts per day than the other two rehabilitation groups (Fig. 1). The only rehabilitation parameter that was not significantly different across rehabilitation conditions was running velocity, largely due to high individual variance (Fig. 1 and supplementary material Fig. 1). Both the low distance and restricted rehabilitation groups rested for significantly more hours per day than the unrestricted rehabilitation group throughout all weeks of rehabilitation (Fig. 1 and supplementary material Fig. 1).

**FIG. 2.**
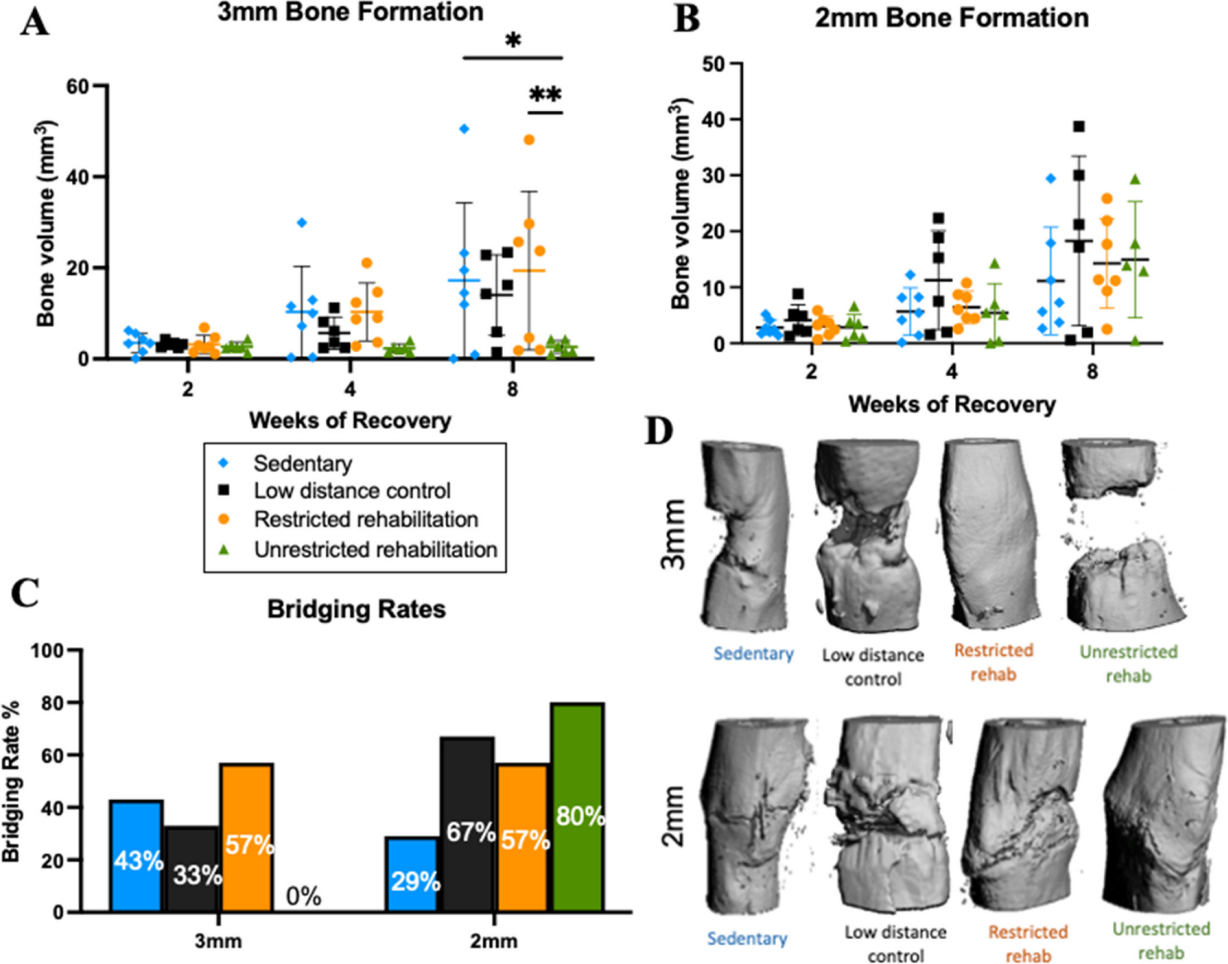
The influence of rehabilitation on bone healing depends on defect size. (a) Bone volume within centered 2.5 mm of 3 mm defects after 8 weeks of recovery. ^*^p = 0.0203 for sedentary vs unrestricted rehab at week 8, two-way ANOVA with Sidak's multiple comparisons test. ^**^p = 0.0053 for restricted rehab, housed out of cage vs unrestricted rehab at week 8, two-way ANOVA with Sidak's multiple comparisons test. (b) Bone volume within centered 1.5 mm of 2 mm defects after 8 weeks of recovery. (c) Endpoint bridging rates based on radiograph and microCT scans. (d) *In vivo* representative radiographs reveal nonunion for the unrestricted rehab group and relatively bridging integrity for the other experimental groups. Mean ± standard deviation for all graphs.

Longitudinal bone volume quantifications by *in vivo* microCT scans revealed that rehabilitation conditions did not significantly influence average group bone healing of 2 mm segmental bone defects [Fig. 2(b)]. In contrast, bone healing after 3 mm defects was significantly impacted by rehabilitation conditions [Fig. 2(a)]. Unrestricted rehabilitation, which resulted in animals training more often and resting for fewer hours each day, significantly reduced bone formation compared to restricted rehabilitation (p = 0.0053) and sedentary counterparts (p = 0.0203). Bridging rates depended on injury severity, as the unrestricted rehabilitation group exhibited an 80% bridging rate for 2 mm defects but a 0% bridging rate for 3 mm defects [Fig. 2(c)]. Representative microCT 3D reconstructions of 3 mm defects revealed nonunion with clear absence of bone formation across all animals in the unrestricted rehabilitation group, while the restricted rehabilitation group exhibited complete union across 57% of animals [Figs. 2(c) and 2(d)].

**FIG. 3.**
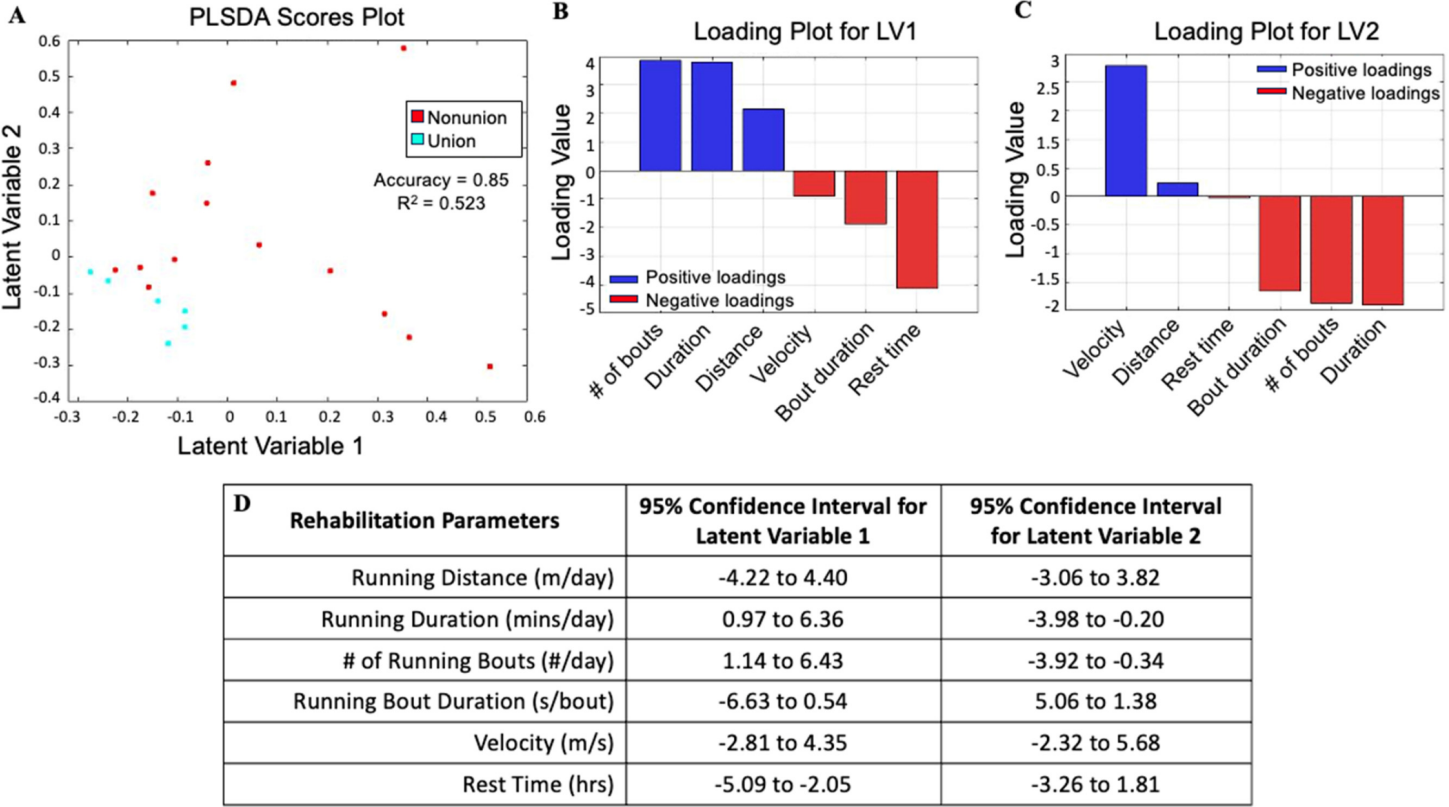
PLSDA of activity data across weeks 1–3 of rehabilitation on bone healing outcomes exhibits poor model fit. (a) PLSDA analysis established latent variable 1 (LV1) and latent variable 2 (LV2) to explain healing outcomes via rehabilitation parameters. (b) and (c) Latent variable loading plots visualize the contributions of each rehabilitation parameter to bone healing outcomes. Blue bars represent positive correlation. Red bars represent negative correlations. (d) Table of the 95% confidence interval across each rehabilitation variable within LV1 and LV2 using bootstrapping.

To help distinguish between unions and nonunions based on rehabilitation parameters, we performed partial least squares discriminant analysis using activity data (running distance, running duration, running velocity, running bouts/day, running bout duration, and rest time) and end point bridging status (1 = union and 0 = nonunion) determined by microCT scans. This linear regression analysis demonstrated poor separation across latent variable 1 (LV1) and latent variable 2 (LV2) and a moderate R-squared (Fig. 3). The latent variables are combinations of the measured rehabilitation variables with loadings that correspond to variable importance on end point bone volume. We also used bootstrapping. The confidence intervals for LV1 and LV2 revealed overlap, and both latent variables did not explain much of the variance [Figs. 3(b) and 3(c)]. For example, the 95% confidence interval for running distance was −4.2 to 4.4 for LV1 and −3.06 to 3.82 for LV2, indicating a lack of separation across latent variables.

**FIG. 4.**
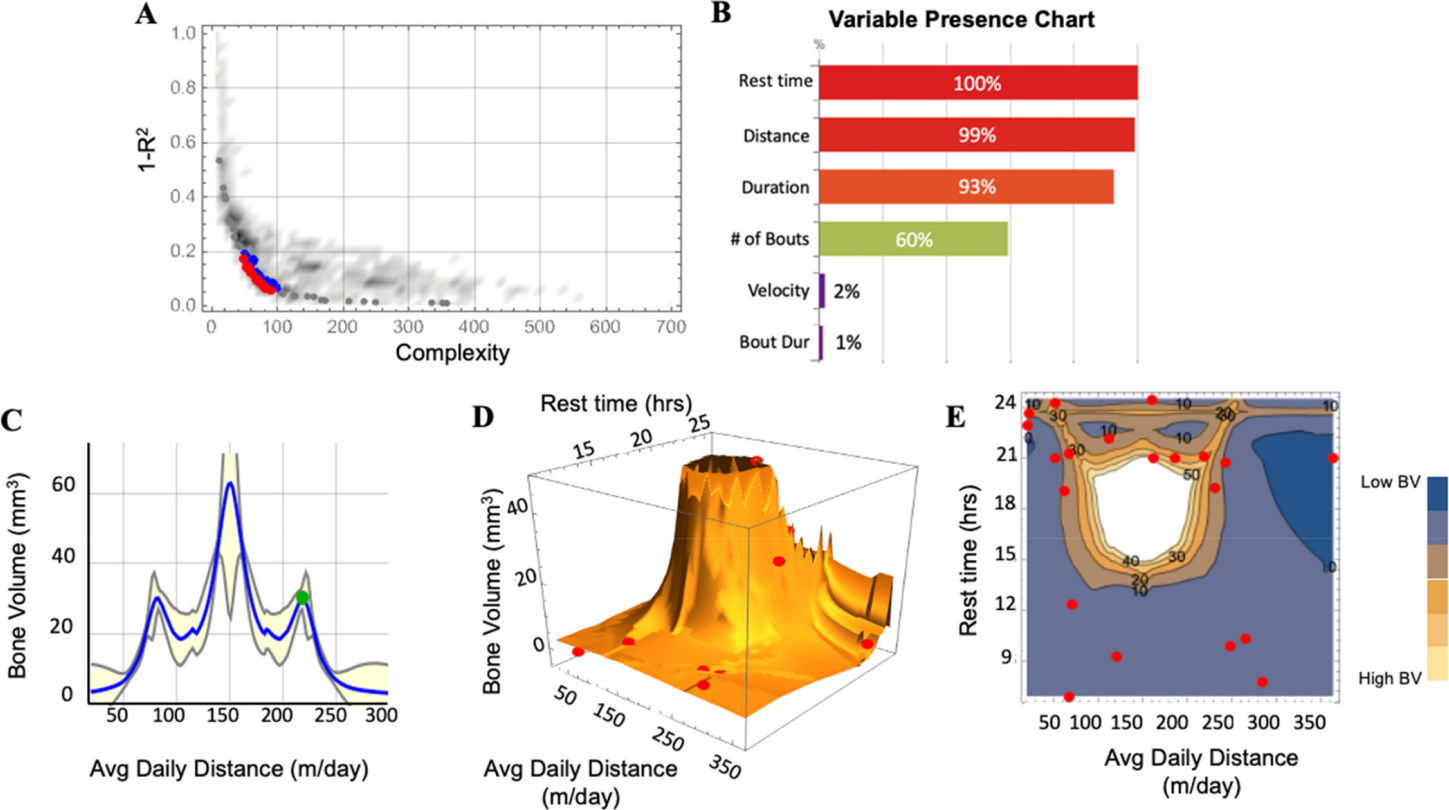
Nonlinear multivariate models support early restricted rehabilitation paired with longer rest times to improve bone formation. (a) The top models were selected at the “knee” of the Pareto front, representing the most accurate models with the lowest degree of complexity. (b) The variable presence chart reveals the percentage of models that contained each rehabilitation parameter. (c) 2D response profile plots revealing the relationship between daily average distance and bone volume across models that only include max rest, running distance, and running duration variable combinations. The blue line represents the predictive model ensemble, and the yellow envelope demonstrates the variance in the ensemble as a function of each variable. (d) and (e) 3D response surface plot and 2D contour plot, respectively, illustrate bone volume as a function of rest time and average daily distance. Red datapoints indicate individual animals.

AI-based genetic programming algorithms were used to perform multivariate regression analyses and generate 1315 unique models to relate rehabilitation parameters with end point bone healing (Evolved Analytics, DataModeler software). Similar to our previous study, 108 models from the “knee” of the Pareto front were selected to investigate models with less complex mathematical equations (i.e., a small number of parameters, basic mathematical operations, and minimal interactions between variables) and high accuracy scores [Fig. 4(a)].^47^ Rehabilitation distance, duration, and rest time were present in nearly all 108 of the “fittest” models, suggesting that these parameters are likely influential to bone formation [Fig. 4(b)]. Our multivariate regression analyses included variables that were dependent on each other (e.g., running duration is directly related to running bout duration and the number of running bouts per day). Therefore, feature selection was performed to only include independent variables that were present in models with high accuracy and low complexity (distance, duration, and rest time) for the nonlinear evaluation of rehabilitation parameters and bone healing [Figs. 4(a) and 4(b)].

After feature selection, we were able to visualize the nonlinear relationship between rehabilitation distance and bone healing [Fig. 4(c)]. Response plots, which visualize predicted bone volume response to rehabilitation inputs, were used to identify how input variables relate to end point bone healing. Our plot revealed a distinct nonlinear relationship between average daily running distance and end point bone volume [Fig. 4(c)]. Three-dimensional surface plots also revealed a distinct nonlinear relationship between the effect of average daily running distance and rest time on end point bone volume [Fig. 4(d)]. Data from all animals with the same bone volume were connected to produce contour lines to visualize the multifactorial influence of rehabilitation distance and rest time on bone volume [Fig. 4(e)]. Overlay of individual datapoints (in red) on the 3D surface plot and 2D contour plot illustrated that our dataset lacks animals that ran a moderate amount of daily distance and rested for a moderate number of hours [Fig. 4(e)]. The 2D response plot further illustrated this region of uncertainty with a greater yellow envelope, which corresponded to model variance, around the curve peak [Fig. 4(c)]. However, the model predicted improved bone healing within this region since the regions surrounding it were well represented with increasing amount of bone volume [Figs. 4(c) and 4(e)].

**FIG. 5.**
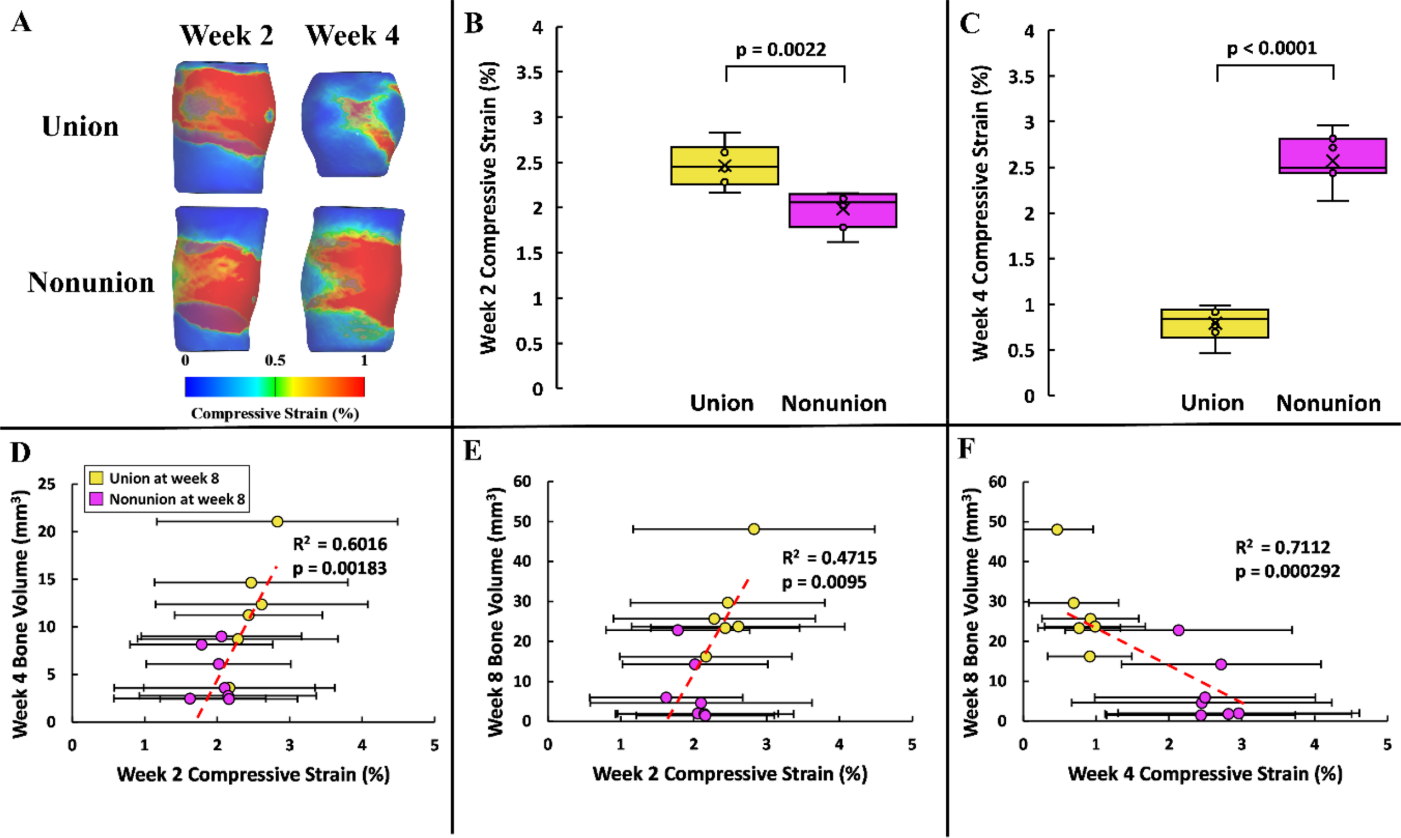
Influence of local compressive strain distribution on end point bone volume. (a) Local compressive strain distribution at the regenerative niche is shown for two representative subjects at weeks 2 and 4 post-injury. (b) and (c) Union subjects exhibited significantly different compressive strain compared to nonunion subjects at both weeks 2 and 4. (d) and (e) Subjects with higher compressive strain at week 2 demonstrated greater bone volume at weeks 4 and 8. (f) A negative correlation was observed between compressive strain at week 4 and bone volume at week 8, contrasting the positive trend seen with week 2 compressive strain.

The local distribution of compressive strain was assessed within the regenerative niche of individual subjects at weeks 2 and 4 post-injury using finite element analysis [Fig. 5(a)]. Our objective was to investigate whether early strain influenced healing outcomes by quantifying week 2 and week 4 defect strains for subjects that exhibited union vs nonunion after 8 weeks of recovery. At week 2, the average compressive strain was significantly higher for union subjects compared to nonunion subjects (p = 0.0022) [Fig. 5(b)]. Interestingly, the trend reversed at week 4, where union subjects exhibited significantly lower average compressive strain compared to nonunion subjects (p < 0.0001) [Fig. 5(c)]. Moreover, subjects with greater average compressive strain in the regenerative niche at week 2 demonstrated higher bone volume at weeks 4 and 8 than those with lower compressive strain (R^2^ > 0.45; p < 0.01) [Figs. 5(d) and 5(e)]. On the other hand, subjects with increased bone volume after 8 weeks of rehabilitation experienced lower average compressive strain at week 4 (R^2^ = 0.7112; p = 0.000 29) [Fig. 5(f)]. We observed a very similar pattern for shear strain distribution. Similar to compressive strain, shear strain at week 2 was higher in union subjects compared to nonunion subjects (p = 0.0032), and this relationship reversed at week 4 (p < 0.0001). We also found that higher shear strain at week 2 was associated with greater bone volume at weeks 4 and 8 (R^2^ > 0.45), whereas higher shear strain at week 4 was linked to lower bone volume at week 8 (R^2^ = 0.7155; p = 0.000 269) (supplementary material Fig. 2).

We also quantified the average compressive strain within the mineralized and non-mineralized tissues at the regenerative niche and regressed those values against bone volume quantification at distinct time points. Our regression included week 2 strain vs week 4 bone volume, week 2 strain vs week 8 bone volume, and week 4 strain vs week 8 bone volume. The week 2 compressive strain in mineralized tissues showed a random relationship with future bone volume, as indicated by the weak correlation with bone volume at weeks 2 and 4 (R^2^ < 0.15; p > 0.2) [supplementary material Figs. 3(a) and 3(b)]. However, the compressive strain in mineralized tissues at week 4 showed a stronger correlation with week 8 bone volume than the week 2 strain data (R^2^ = 0.4909; p = 0.0076) [supplementary material Fig. 3(c)]. For the non-mineralized tissues, the average compressive strain showed a strong correlation with bone volume at later time points (R^2^ > 0.5; p < 0.05) [supplementary material Figs. 3(d) and 3(f)]. This correlation largely accounted for the overall relationship observed when analyzing strain across all tissues within the defect [Figs. 5(d) and 5(f)]. Notably, the correlation trend shifts with time as subjects showing higher bone volume at week 8 exhibited greater compressive strain at week 2 and lower strain at week 4 [supplementary material Figs. 3(e) and 5(f)].

**FIG. 6.**
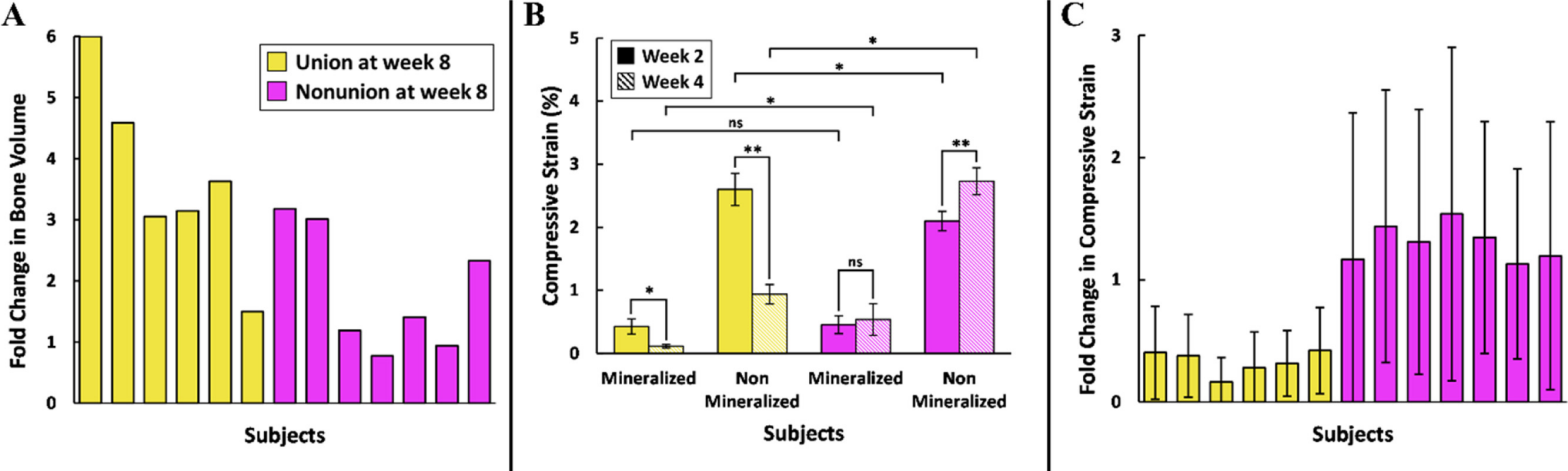
Temporal changes in compressive strain from week 2 to week 4 as an indicator of defect healing progress. (a) No consistent patterns were observed in the temporal changes in bone volume based on the final healing outcome. Nearly all subjects exhibited increased bone volume from weeks 2 to 4, regardless of their healing outcomes by week 8. (b) Significant differences were observed in the compressive strain of mineralized and non-mineralized tissues based on their healing outcomes (^*^p < 0.01; ^**^p < 0.001; and ns = not significant). For union subjects, compressive strain decreased in both mineralized and non-mineralized tissues from weeks 2 to 4. In contrast, nonunion subjects showed either no change in strain over time (mineralized tissues) or an increase in strain (non-mineralized tissues). (c) All six union subjects exhibited a reduction in average compressive strain at the regenerative niche between weeks 2 and 4, while nonunion subjects maintained elevated compressive strain at week 4 post-injury.

Fold changes in experimentally measured bone volume and compressive strain of each limb from week 2 to week 4 were assessed (Fig. 6). The change in bone volume at the regenerative niche did not have a clear association with end point bridging rates since subjects that had a similar increase in bone volume exhibited end point union and nonunion due to bone formation not always bridging the defect gap. As expected, bone volume increased by 1.2 to sixfold for all subjects that achieved union by week 8 [Fig. 6(a)]. Unexpectedly, bone volume also increased for all but two subjects that exhibited nonunion by week 8 due to bone formation not bridging the entire defect region [Fig. 6(a)]. However, comparing changes in compressive strain for mineralized and non-mineralized tissues separately, revealed a marked temporal disparity between subjects who achieved union after 8 weeks and those who did not [Figs. 6(b) and 6(c)]. For union subjects, compressive strain significantly decreased in both mineralized (p < 0.01) and non-mineralized (p < 0.001) tissues [Fig. 6(b)]. In contrast, the compressive strain in mineralized tissues of nonunion subjects remained relatively unchanged over time, while non-mineralized tissues exhibited higher strain at week 4 compared to week 2 (p < 0.001) [Fig. 6(b)]. Additionally, the average compressive strain in non-mineralized tissues at weeks 2 and 4 differed significantly between union and nonunion subjects (p < 0.01) [Fig. 6(b)]. No significant difference in compressive strain in mineralized tissues was observed between union and nonunion subjects at week 2. However, by week 4, the strain was significantly lower in union subjects (p < 0.01). Looking at mineralized and non-mineralized tissues together in the regenerative niche, the average compressive strain decreased by a factor of 0.17–0.42 for the union subjects [Fig. 6(c)]. Conversely, the mean compressive strain at the regenerative niche in nonunion subjects increased by 1.12- to 1.54-fold by week 4, indicating that the absence of bony callus formation contributes to persistently elevated local strain magnitudes [Fig. 6(c)].

**FIG. 7.**
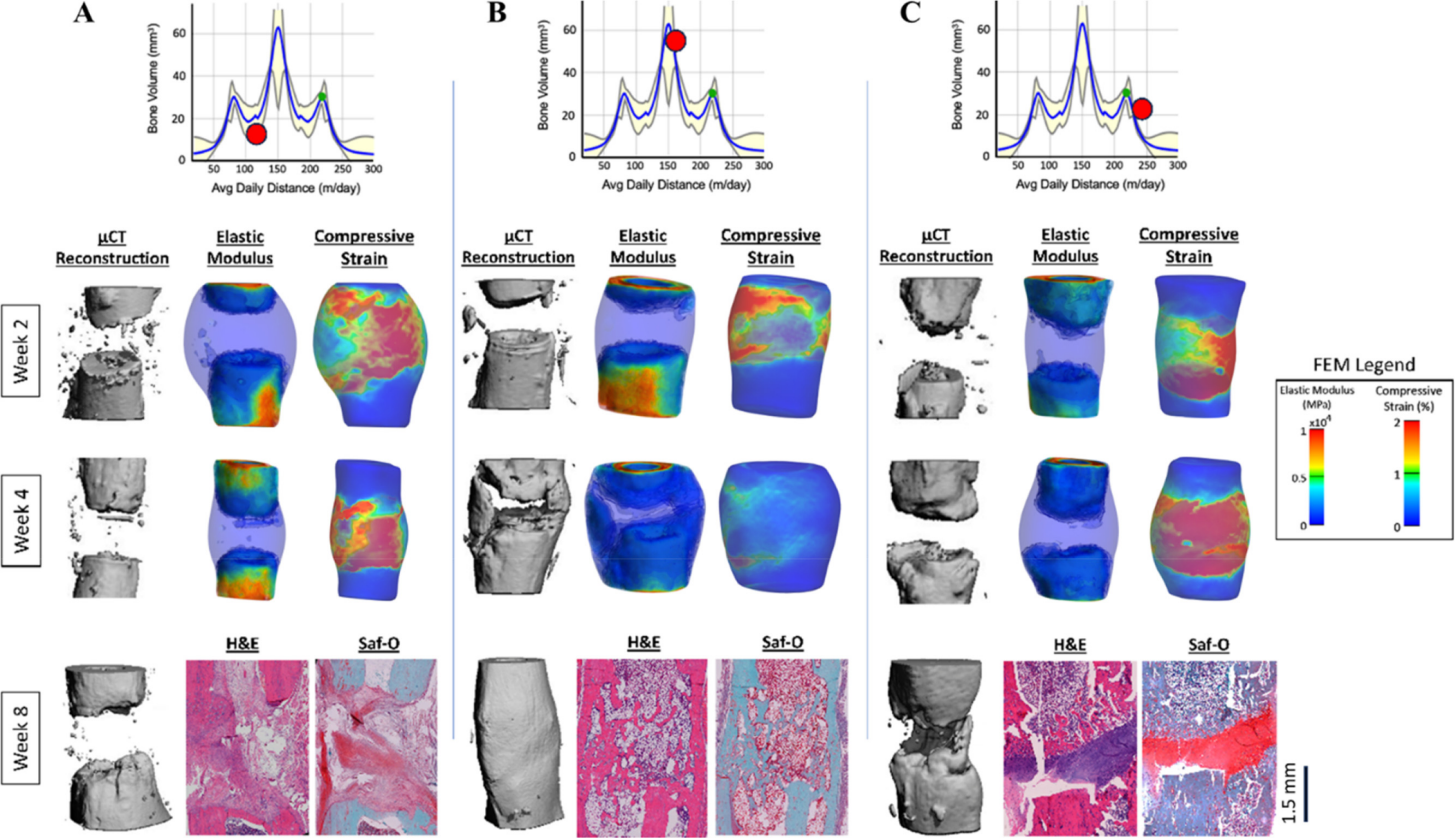
Subject-specific holistic understanding of rehabilitation distance and longitudinal 3 mm defect local niche mechanics and healing outcomes. (a) Subject-specific data column with low daily running distance. (b) Subject-specific data column with near-optimal running distance. (c) Subject-specific data column with high daily running distance. Subject-specific activity data are shown via red dots on the response plot (bone volume vs average daily distance) across the top row.

Since we collected subject-specific injury geometry, activity, and strain data, we wanted to examine subject-specific longitudinal data to better understand the effects of rehabilitation on local mechanics and bone healing. Our study enabled the tracking of subject-specific longitudinal healing status via 3D microCT reconstructions, regenerative niche mechanics via FE models, and end point tissue phenotype via histology (Fig. 7). Subject-specific data revealed that a subject that ran less than the predicted optimal running distance resulted in nonunion with high levels of compressive strain throughout weeks 2 and 4 and fibrotic tissue within the defect region by week 8 [Fig. 7(a)]. A subject that ran more than the predicted optimal running distance also resulted in nonunion with high levels of compressive strain throughout weeks 2 and 4 post-injury and a thick cartilaginous band spanning the defect region by week 8 [Fig. 7(c)]. Finally, a subject that ran a near-optimal amount of daily running distance had local strains that decreased in magnitude overt ime and uniform bridging with signs of remodeling throughout the defect region by week 8 [Fig. 7(b)].

## DISCUSSION

Mechanical loading strategies to improve bone healing have largely investigated loading magnitude as the dominant parameter dictating the regenerative response by modifying fixation plate stiffness^5,6,9,10,12,27–29^ or applying controlled external loads.^20–26^ These studies often involved the controlled investigation of one or two variables at a time, even though clinical rehabilitation is inevitably multifactorial. The present study examined the influence of three rehabilitation profiles with six distinct rehabilitation parameters (running distance, running duration, running velocity, number of running bouts, running bout duration, and rest) on segmental bone healing. Our injury model involved bilateral 2 and 3 mm segmental bone defects in rodent femora that were stabilized with compliant fixation plates and not treated with biologics (n = 5–7, N = 25 rats, totaling to 50 segmental bone defects). Bilateral defects were utilized to reduce the number of animals for this study and the potential for animals to shield their injured limb, minimizing the variability of the load experienced by the bone injuries within an experimental group. To create distinct post-injury rehabilitation conditions, animals were either left in sedentary conditions or given unrestricted wheel access (24/7) or restricted (2 h/day). Bone healing, running wheel activity data, and subject-specific FE models of the regenerative niche were longitudinally monitored, and the relationship between rehabilitation parameters and bone healing was assessed via multivariate linear and nonlinear regression analyses. This experimental setup allowed us to investigate the combined influence of six different rehabilitation variables to understand (1) which variables were important to bone healing and (2) what relative level of these variables should be targeted to improve bone healing.

Within this study, we examined three rehabilitation conditions to examine the combined effect of rodents' wheel running duration, running distance, the number of running bouts, running bout duration, running velocity, and daily rest time on bone healing (Fig. 1 and supplementary material Fig. 1). We found that rodent wheel access (2 vs 24 h per day) and housing conditions (with or without running wheel in rodent's home cage) resulted in three distinct rehabilitation profiles that can be summarized as a (1) low activity profile, (2) frequent activity with little rest, and (3) moderate activity paired with longer daily rest, which, respectively, corresponded to the low distance control, unrestricted rehabilitation, and restricted rehabilitation groups (Fig. 1). Running activity was limited for the low distance control and restricted rehabilitation groups through different wheel protocols: the low distance control group by cage transfers and the restricted rehabilitation group by braked wheels. The impact of these unique rehabilitation profiles on segmental bone defect healing depended on injury size. More specifically, unrestricted rehabilitation resulted in 20% nonunion for smaller 2 mm defects and 100% nonunion for larger 3 mm defects (Fig. 2). Our results ultimately suggest that for more severe injuries, restricting exercise and ensuring adequate rest is critical to avoid negative effects on bone healing, such as disruption of the vasculature or excessive interfragmentary movement. However, it is worth noting that these results are in the context of one defect stabilization method. Previous work in our 6 mm rodent segmental defect model investigated the influence of fixation plate stiffness on bone healing. They found that fixation plates with higher flexural stiffness resulted in decreased local strains along the fixation plate and within the defect region, and ultimately resulted in impaired healing.^5^ In the context of our current study, changing the fixation plate stiffness would likely impact the necessary levels of activity and rest needed for improved bone healing. For example, a flexible fixation plate may require less daily distance and longer daily rest periods since the fixation plate would share more of the ambulatory load with the healing callus. Previous work has shown that fixation plates that are too flexible (axial stiffness = 8.4 ± 0.4 N/mm) can significantly impair healing.^9^ On the other hand, fixation plates that are too stiff might also mitigate the influence of rehabilitation on bone healing by shielding too much of the ambulatory loads.

Both the 2 and 3 mm defects exhibited relatively modest bridging rates of 29% and 43%, which are expected since these segmental defects are close to previously established critically sized femoral defects in Wistar rats.^48,49^ Poser *et al.* found that 5 mm femoral middiaphyseal defects were critically sized when stabilized with polyetherketone (PEEK) internal fixators for 15-week-old female Wistar rats (body weight ∼184 g).^48^ The rats used by Poser *et al.* were lighter than the rats in this study (275–325 g). Although the authors did not list the axial stiffness of their PEEK fixation plates, we know that PEEK is a stiffer material than polyethylene, so we can infer that our fixation plates are less stiff. With both the lighter rat weights and stiffer fixation stiffness, defects investigated by Poser *et al.* likely experienced lower magnitude loading than this study, due to less load-sharing between the fixation plate and healing callus. Harrison *et al.* found that 3 mm femoral middiaphyseal segmental defects were critically-sized when stabilized with external fixators for 12-month-old male Wistar rats (body weight 450–550 g).^49^ The rats investigated by Harrison *et al.* were heavier than the rats in this study. The external fixators were also less stiff (∼45–46 N/mm) than our fixation method (∼145 N/mm). The size of a critically-sized defect has been shown to depend on the rat strain, weight, age, sex, and fixation method.^50^ Since our internal fixation plates exhibit a stiffness between these two studies, it is reasonable to expect the critical size for our rodent model to fall somewhere between 3 and 5 mm. In fact, radiographs and microCT scans from a previous pilot study (n = 2–4) revealed that 2 and 3 mm defects resulted in a portion of defects bridging within the 8-week timeframe while 4 mm defects exhibited no bridging with minimal bone formation (supplementary material Fig. 4). For the current study, we utilized 2 and 3 mm defects because this model enables the detection of a potential improvement with rehabilitation without the use of biologics and their known confounding factors.

The linear regression analysis produced a model with moderate goodness of fit (R^2^ = 0.524) and poor separation along both latent variables, suggesting that the influence of rehabilitation on bone healing cannot be adequately described with this approach (Fig. 3). Previous literature has suggested a nonlinear relationship between loading magnitude and bone healing, where moderate loading magnitude improved bone formation, but high magnitude loading was severely detrimental.^5,9^ This motivated a nonlinear modeling approach, allowing us to elucidate multifactorial, nonlinear relationships between additional rehabilitation parameters and bone healing.

Several researchers have investigated advantageous vs detrimental loading conditions for bone healing. Previous studies by Rubin *et al.* have found that osteogenic responses are predominantly influenced by strain magnitude, strain rate, and frequency.^51–56^ Loading duration was also found to influence bone tissue, with extended loading periods having a diminishing effect.^51^ However, these studies investigated adaptations of intact bones to loading since they did not involve an injury model. In addition, several groups have investigated interfragmentary motion and its clinical implications, including rehabilitation regimens.^28,31,35,36^ Both excessive and inadequate interfragmentary motion can negatively influence bone healing. The mechanical environment of a healing fracture is determined by the stiffness of fixation and weight bearing, suggesting that fracture healing requires a balance of both fixation and rehabilitation.^8,27,57^ Flexible fixation strategies should not be paired with intense rehabilitation, just like rigid fixation strategies should not be paired with conservative rehabilitation. Although these studies and others have advanced mechanobiology principles, they did involve subject-specific tracking of multiple rehabilitation parameters to discern multifactorial relationships between several rehabilitation parameters and bone healing. In our study, we were able to track subject-specific activity to calculate six rehabilitation parameters used as inputs into multivariate regression analyses. Our results revealed that daily running distance and rest were the most influential rehabilitation parameters on bone healing [Fig. 4(b)]. Nonlinear regressions revealed a distinct nonlinear relationship between average daily distance and end point bone formation, where a moderate amount of daily distance paired with longer rest correlated with improved bone formation [Figs. 4(c) and 4(e)]. These results indicated that rehabilitation decisions must consider the importance of rest, especially since rehabilitation regimens with shorter rest periods resulted in 100% nonunion after larger bone injuries. Additional research to expand our multifactorial understanding of the influence of rehabilitation regimens on bone healing will be crucial to inform clinical rehabilitation decisions. In particular, future experiments will focus on subjects that run a moderate daily distance and rest for a moderate number of hours, as these conditions were under-represented.

Based on mechanobiology principles, we hypothesize that adequate rest time is crucial for the underlying mechanisms of bone healing, especially during the first month after injury. Bone healing consists of the inflammatory stage (0–7 days), followed by soft callus formation (2–3 weeks), hard callus formation (3–4 months), and eventual remodeling.^33^ Our rehabilitation regimens started after the inflammatory stage (day 7), and our results found that daily rest throughout soft callus formation was crucial for larger defects. This stage of healing entails revascularization through angiogenesis and vasculogenesis, progenitor cells (e.g., MSCs) differentiating into fibroblasts and chondrocytes, recruitment of precursor cells, and ossification.^33,58^ The importance of rest throughout these physiological processes is supported by literature that found osteogenic cells can become saturated with dynamic loading after only a few loads.^59^ The addition of rest in between loading cycles has been shown to promote enhanced activation of osteoblasts and increased bone formation.^60–63^ Srinivasan *et al.* investigated whether the insertion of rest could make a non-osteogenic loading magnitude osteogenic. They found that inserting rest significantly increased bone formation (8-fold increase) compared to low-magnitude loading without rest.^64^ Rest also improved osteogenic response in an aged, osteoporotic model.^65^ These studies hypothesized that rest in between loading cycles enhances the osteogenic response by maximizing transient fluid flow, known to stimulate osteogenic cells.^66^ Our results support the importance of rest between exercise sessions and further suggest that larger bone injuries may require more rest than smaller ones.

Balancing activity and rest throughout early rehabilitation is also supported by literature that found excessive loads inhibited vascularization, progenitor cell differentiation, and bone formation.^9,67–69^ Checa and Prendergast used a mechano-biological model to investigate vascular network development and tissue growth inside a bone scaffold under different loading conditions. They found that low levels of mechanical loading stimulated bone formation, while high levels inhibited capillary growth and bone formation.^68^ Bratengeier also found that progenitor cells exhibited an osteodestructive response with high-amplitude fluid flow compared to cells that underwent low-amplitude fluid flow.^69^ Finally, in a segmental defect model, Boerckel *et al.* found that excessive early loading significantly inhibited the vasculature and bone regenerative responses.^9^ Building on these mechanobiology principles, our results suggest that rehabilitation must provide advantageous levels of activity while balancing adequate rest periods to improve bone healing. Our study revealed that the relative levels of activity and rest depended on injury severity since unrestricted rehabilitation resulted in 0% union for larger bone injuries but 80% union in smaller injuries. These results further suggest that bone injuries that are only one millimeter different in size exhibit different mechanosensitivity. Injuries of different sizes are known to heal through different mechanisms because of the different vessel regrowth requirements and local mechanics, which direct endochondral vs intramembranous ossification.^35,70,71^ Larger bone injuries may be more mechanosensitive because of their increased instability, greater interfragmentary movement, reduced progenitor pool, and greater damage to the vasculature compared to smaller injuries.^35,72–76^ It is also worth noting that daily rest is inversely related to the number of running bouts/day (i.e., as daily rest increases across the different rehabilitation profiles, the number of daily running bouts decreases). Therefore, larger defects, with greater mechanosensitivity, may benefit from greater rest because this rehabilitation regimen also ensures fewer running bouts each day. Conversely, the stability and smaller interfragmentary movement of smaller defects may enable these defects to withstand and even benefit from more frequent loading. This theory is supported by the highest bridging rate (80%) seen in the 2 mm defects that underwent rehabilitation with the shortest daily rest and the highest number of running bouts (unrestricted rehabilitation).

Our multivariate nonlinear regression models support restricted early running distance and longer daily rest. However, a limitation of our dataset is that it is small for training a predictive model (N = 25 rats). Instead, our model was intended to identify influential rehabilitation parameters and their respective relationship to bone healing. To develop a robust predictive model, our dataset would require a larger sample size to better represent the entire range of daily running distance and rest time potentials. It is also worth noting that our work was performed in near-critically sized bone defects without the use of biologics. Future work could investigate rehabilitation in a critically-sized injury treated with growth factors or other secondary treatments to simulate a broader range of clinical scenarios.

The novel FE analysis pipeline employed in this research facilitated the application of subject-specific geometry and material assignment for both the defect and ectopic bone, combined with a generic model of the proximal and distal femur and fixation plate. The irregular shape observed in the ectopic bone formation surrounding the defect [Fig. 5(a)] underscores the importance of utilizing a subject-specific defect geometry rather than an assumed shape (e.g., a cylinder).^77^ Furthermore, by incorporating the trabecular bone and marrow regions, we enhanced the accuracy of the generic femur model compared to previous studies.^78,79^ By accurately representing the geometry and material properties of the femur and healing region, we characterized the interaction between mineralization and the local mechanical environment and their influence on bone healing. A minor limitation of this study is the assumption of isotropic material symmetry for the femur rather than orthotropic symmetry. In our simulations, the magnitude of compressive strain in the defect region is much higher than in the cortical bone, typically with a ratio of about 25:1. Due to the very small relative deformation experienced by the cortical bone segments, the simplifying assumption of isotropy for cortical bone was reasonable.^80^ FE analysis is a valuable tool for determining the optimal compressive strain range to facilitate fracture healing. Importantly, we utilized experimentally measured strain data to inform the boundary conditions, one of the most crucial input parameters for accurately measuring local strain at the defect. Subject-specific local strains are a function of load transfer through the defect region and the individualized amount of bone distribution within the defect. In this study, we created subject-specific finite element models using bone formation in the defects but applied the same representative boundary conditions from historical fixation plate strain data.^46^ Although the time course of fixation plate strains during healing follows a consistent pattern between animals, fully subject-specific analyses in the future should include both individualized bone formation and defect boundary conditions. Finally, to the best of our knowledge, this is the first FE workflow to separately analyze the influence of mineralized and non-mineralized tissues on the bone healing process.

Local compressive strain demonstrated a strong correlation with defect healing progression. Existing literature indicates that for humans, maintaining compressive strain within 2%–10% at the fracture site supports healing by promoting angiogenesis and osteogenesis while suppressing osteoclast differentiation and bone resorption.^33^ However, recent studies have shown that the “2%–10% rule” might not be universal, and the optimal strain range depends on the severity of the fracture and the species and size of the subject.^81,82^ In addition, with healing time, the interfragmentary strain changes depending on the degree of mineralization, which also changes the optimal strain for different time points.^11^ In our study, we observed that at week 2, the average compressive and shear strains at the defect was higher than 1% for all subjects [Fig. 5(b); supplementary material Fig. 2(b)]. With time, as mineralization progressed for the union subjects, the average strains dropped below 1%, indicating redistribution of the mechanical load more uniformly across the healing tissues [Fig. 5(c); supplementary material Fig. 2(c)]. Previous work has suggested that “reverse dynamization,” involving higher strain early followed by reduction during healing, yields significantly better outcomes for near-critical segmental defects than the conventional “dynamization” approach, where compressive strain at the defect region is progressively increased over time.^83^ The high strain at the early stages of healing is important for forming a soft callus (cartilage), which stabilizes the fractured region.^83^ Afterward, the stable environment facilitates the mineralization of the cartilage extracellular matrix into a bony callus through endochondral ossification, ultimately forming mature bone.^84,85^ In addition, the low-strain environment is optimal for mesenchymal stem cells (MSCs) to differentiate into osteoblasts, enhancing the likelihood of achieving union.^86^ However, sustained high compressive strain may inhibit the ossification process, which hinders bony callus formation and leads to nonunion.^34,35^ This is supported by subjects with high compressive and shear strains, even after week 4, achieving low bone volume after 8 weeks of rehabilitation in this study (p < 0.001) [Fig. 5(f); supplementary material Fig. 2(f)].

The compressive strain of mineralized and non-mineralized tissues is key to the mechanoregulation of bone healing, with its timing and location shaping tissue development. Mineralized and non-mineralized tissues react differently to similar loading conditions.^87^ The mineralized tissues are more dense, stiffer, and less sensitive to mechanical stimulus than non-mineralized tissues.^88^ The load on the mineralized tissues at week 2 may not be large enough to trigger remodeling, which may explain the negligible correlation between the compressive strain of the mineralized tissues at week 2 and bone volume at later times [supplementary material Figs. 3(a) and 3(b)]. However, as healing progresses and the amount of mineralized tissues at the regenerative niche increases, the load gets evenly distributed.^89^ The redistribution of load may have resulted in the reduced compressive strain in the mineralized tissues of the union subjects at week 4 compared to week 2 [supplementary material Fig. 3(c)]. On the other hand, the deformation of the less dense, non-mineralized tissues under optimal load stimulates the cells to differentiate and proliferate, accelerating the formation of new bone.^86^ The strong positive correlation between week 2 compressive strain in non-mineralized tissues and week 4 bone volume highlighted the sensitivity of the non-mineralized tissues to mechanical cues [supplementary material Fig. 3(d)]. Although the correlation trend persisted between week 2 strain and week 8 bone volume, the mineralization and remodeling process over the two weeks might have reduced the dependency, resulting in a slightly weaker correlation [supplementary material Fig. 3(e)]. On the other hand, the lower week 4 compressive strain of the non-mineralized tissues correlating with the higher week 8 bone volume is likely the result of the mineralized tissues carrying the majority of the loads [supplementary material Fig. 3(f)].^89^ For the nonunion subjects, due to the lack of load distribution in the mineralized tissues, the strain remains high for the non-mineralized tissues. By separately evaluating strain in mineralized and non-mineralized tissues, we could explain why we observed the shift in optimal strain in time for the union subjects and how the load distribution of the mineralized tissues influences the healing outcome.

Relying solely on bone volume extracted from microCT may not suffice to characterize union and mechanical strength recovery.^90^ Moreover, the early healing periods often do not involve mineralization changes detectable by radiographic imaging, making it difficult to assess healing and mechanical stability from radiography alone.^91^ In our study, almost all of the subjects exhibited a two- to sixfold increase in bone volume from week 2 to week 4 post-injury [Fig. 6(a)]. Yet, in more than half of the subjects, union did not occur in the regenerative niche by week 8. However, an increase in compressive strain with time was a distinct characteristic of all nonunion subjects [Figs. 6(b) and 6(c)]. This aligns with previous studies, which experimentally observed the sustained high strain at the defect region for subjects with nonunions, as the absence of bony callus hindered load sharing and redistribution.^91^ Although there was new bone deposition in nonunion subjects according to the increased bone volume, the lack of mineralized tissue bridging the defect might have caused the sustained high compressive strain in the mineralized tissues at week 4 [Fig. 6(b)]. On the other hand, the significant decrease in compressive strain in the mineralized and non-mineralized tissues indicated union after 8 weeks [Fig. 6(b)]. As discussed previously, the local mechanical environment is significantly different at weeks 2 and 4 depending on whether subjects achieved union or nonunion for proper load redistribution. Despite having femoral defects of similar size, differences in rehabilitation regimens and the resulting compressive strain at the regenerative niche determined whether union would occur after eight weeks. Although the bone volume data were inconclusive, we can confidently say, that a reduction in average strain from week 2 to week 4 is a representative attribute of subjects that achieved union [Fig. 6(b)]. FE analysis provided detailed insight that could not be obtained from micro-CT scans or radiographs. For large femoral defects, factors, such as defect size, alignment, and limitations in bone ingrowth and mechanical strength recovery, necessitate individualized approaches.^46^ While generic rehabilitation protocols may be adequate for smaller fractures, tailored regimens can optimize recovery, reduce risks, and improve long-term outcomes for more severe injuries.^92^

Clinical prescriptions of rehabilitation that account for subject-specific factors have the potential to improve clinical efficacy. Evidence-based rehabilitation guidelines depend on technology and research that enables subject-specific analysis to better explore rehabilitation as a therapeutic approach for bone healing. Our study leveraged subject-specific FE modeling and multivariate models to reveal a holistic and longitudinal understanding of how rehabilitation conditions relate to healing status and niche mechanics (Fig. 7). It is also well known that the local mechanical environment is influenced by surgical stabilization parameters (e.g., fixation method, fixation plate material, etc.) and rehabilitation parameters. However, technology to longitudinally track local subject-specific parameters has been limited. Our subject-specific FE modeling, based on *in vivo* microCT scans form the current study and strain data from earlier studies using our implantable sensor technology, provides a tool to further investigate how additional aspects of bone injuries impact healing outcomes, such as stabilization method (e.g., external fixation vs internal fixation or across different fixator designs). Continued investigation of ad lib post-injury rehabilitation across subjects that undergo various fracture stabilization methods could further elucidate the interaction between loading magnitude and the necessary levels of activity and rest to achieve improved bone healing outcomes.

## CONCLUSION

Our results reveal the critical importance of data-informed and subject-specific rehabilitation decisions to improve the efficacy of rehabilitation regimens after bone injuries. Using our implantable sensor technology and subject-specific FE modeling, we have been investigating an overall hypothesis that bone healing can be improved by achieving local strains within beneficial ranges that change as a function of time. For this study, we further hypothesized that this beneficial window of strain would change as a function of injury severity. However, our results revealed a more complicated reality: bone healing depends on more than an advantageous magnitude of local strains. Instead, bone healing also depends on an adequate balance between activity and rest that changes as a function of injury severity. Subject-specific rehabilitation decisions for bone healing must consider subject-specific factors, such as injury stabilization, injury severity, and healing status while also enforcing adequate rest. Future work that leverages subject-specific approaches can help advance mechanobiology principles as well as evidence-based rehabilitation to improve clinical outcomes after musculoskeletal injuries.

## METHODS

### Surgical procedure

As previously described, 17-week-old female Wistar (Envigo) rats that weighed between 275 and 325 g received bilateral femoral 2 and 3 mm segmental bone defects.^10^ This study involved 28 animals, totaling 56 segmental bone defects. After defects were made, femora were stabilized with compliant (Ultra-High Molecular Weight Polyethylene) internal fixation plates with a flexural stiffness of 145.2 ± 10.6 N/mm based on previous characterization.^5,93^ Defects were left empty, and animals were randomly allocated to experimental groups with n = 7 animals per group, meaning n = 14 defects per group (sedentary control, restricted rehabilitation, and unrestricted rehabilitation). Due to unexpected similarities in running distance between the restricted and unrestricted rehabilitation groups, we later added a low distance control group that underwent identical surgical conditions. Immediately prior to surgery, rodents were given a subcutaneous dose of 1 mg/kg of Sustained-Release Buprenorphine based on their pre-operative weight and 3 ml of Lactated-Ringer solution (VetOne). An additional 2 ml of Lactated-Ringer solution was given subcutaneously every 24 h throughout the first 3 days of recovery. 48 h after surgery, rodents were given standard Buprenorphine subcutaneously at a dose of 0.03 mg/kg. Postoperative complications, such as hardware failure, were considered exclusion criteria and resulted in early euthanasia (low distance control: n = 1 and unrestricted rehabilitation: n = 2), bringing the total number of animals included in our analyses to 25 animals and 50 segmental bone defects (n = 5–7 defects per group). Animals that did not experience complications were euthanized by CO_2_ asphyxiation at 8 weeks, and representative femora were harvested for histology. All procedures were approved by the University of Oregon IACUC (Protocol #20-32).

### Voluntary treadwheel running

Prior to surgery, rats were pre-trained for a 14-day period where they had voluntary access to in-cage running wheels (Exotic Nutrition, 14″). Running wheels were modified in-house to allow continual activity data collection via revolution counters (Lafayette Instruments^®^). Both the wheels and revolution counters were mounted in standard cage bottoms via 3D-printed parts. After surgery, rats were individually housed in standard cages (Tecniplast) and given 7 days to recover with no wheel access. After recovery, sedentary animals remained in standard cages with no wheel access, and non-sedentary animals followed specific rehabilitation regimens. The restricted rehabilitation group was singly housed in a standard cage and manually transferred to their wheel cages from 8 to 10 pm daily for their 2 h/day of ad lib wheel running. The added low distance control group was singly housed in cages that had a wheel with a brake applied to prevent running until the brake was released from 8 to 10 pm daily. Due to these conditions, subjects in the restricted rehabilitation group were handled more than the low distance control group. Finally, the unrestricted rehabilitation group was individually housed in wheel cages with a wheel that had no brake, allowing 24/7 voluntary running. Cumulative running activity, in the form of distance (meters), was longitudinally monitored using revolution sensors (Scurry Rat Activity Sensor, Lafayette Instruments^®^). A Python script was then used to calculate daily average running distance (m/day), running duration (min/day), number of running bouts per day (bouts/day), velocity (m/s), running bout duration (s), and rest time (h). Running duration was defined as the total daily time (min/day) the rat was considered running on the wheel due to increasing cumulative distance within a 6-s time period; a running bout started once the revolution counter detected a change in cumulative distance and then ended when six consecutive seconds elapsed with no change in cumulative distance; running bout duration was defined as the average time animals spent on the wheel once they started a running bout; and rest time was the maximum daily number of hours that the cumulative distance did not change.

### *In vivo* radiographs and microCT

To monitor bone formation, *in vivo* radiographs and microCT scans were acquired at 2, 4, and 8 weeks post-injury while rats were under anesthesia. Digital radiographs were captured at 40 kV with a 7 s exposure (Faxitron MX-20). MicroCT scans were performed using 48 *μ*m voxels, 55 kVp, 145 mA, and 750 ms integration time (VivaCT 80, Scanco Medical). Trabecular bone formation was evaluated inside a standard 1.5 and 2.5 mm long cylindrical volume of interest (VOI) between the intact bone ends of 2 and 3 mm defects, respectively. The results were reported as longitudinal bone volume.

### Histology

After animals were euthanized, representative femora from all rehabilitation conditions and across both defect sizes were harvested for representative histology (n = 1 across eight groups). Representative animals were selected based on week 8 *in vivo* radiographs. These femora were fixed in 10% Neutral Buffered Formalin (NBF) for 72 h and then transferred into ethylenediaminetetraacetic acid (EDTA) until the bone tissue was completely decalcified. At this point, samples were shipped to Inotiv, Inc. (Boulder, CO) to undergo sample processing and staining. Femora were longitudinally sectioned at 5 *μ*m and stained with Hematoxylin Eosin and Safranin O/fast Green. Histology slides were then imaged on the Leica THUNDER Imager Brightfield at 5× magnification.

### Partial least squares-discriminant analysis (PLSDA)

Activity data averaged across weeks 1–3 of post-injury rehabilitation and end point bridging scores were compiled. Bridging scores included a score of 1 for union and 0 for nonunion. Partial least squares-discriminant analysis (PLSDA) was conducted in MATLAB (MathWorks, R2021b version 9.11) using the Statistics and Machine Learning Toolbox (version 12.2). Data were normalized and scaled prior to running the analysis to eliminate skewed analysis that would be biased toward rehabilitation variables with higher values. For the analysis, the “plsregress” MATLAB function was used to perform the partial least squares regression and then fit a classifier using the latent variables. A scatterplot was then created to visualize latent variables one and two according to their scores. Bar plots were then created to explain each latent variable according to the input rehabilitation parameters and their contributions of these variables to the partial least squares components. The accuracy was calculated using cross-validation with the “kfoldloss” function. The confidence intervals across variables within LV1 and LV2 were calculated using the “bootci” function, and the variance of each latent variable was calculated using the “PCTVAR” output from the “plsregression” function. R-squared was also calculated using the predicted and actual response values.

### DataModeler

Nonlinear multivariate regressions were performed using the DataModeler software (Evolved Analytics) to investigate potential relationships between rehabilitation parameters and end point bone volume. DataModeler uses an evolutionary algorithm for multivariate modeling applications to characterize the nonlinear relationship between inputs and outcomes. DataModeler uses genetic programming to build hundreds of algebraic models and breed “fit” models over successive generations. The fittest models are based on high accuracy and low complexity and aggregate into a predictive model ensemble. This modeling approach provides several advantages over standard regression methods, including (1) the use of nonlinear regression, (2) giving a range for each prediction regime encompassed by the divergence of several models that preserves real statistical uncertainty, (3) stochastically choosing subsets of the records (n's) for each generation, minimizing model bias, and (4) allowing user input where prior knowledge exists (augmented intelligence), such as manual filtering of variable combinations included. Activity parameters were averaged across weeks 1 to 3 of postoperative rehabilitation and regressed against end point bone volume for 3 mm defects. We averaged activity across weeks 1 to 3 of rehabilitation because radiographs and *in vivo* microCT scans confirmed no difference in bone healing until callus formation (week 3 of post-injury rehabilitation). We did not average activity data after the callus formation stage (week 3 of rehabilitation) because of the potential relationship between healing status and activity parameters (e.g., the group with increased bone formation might have greater running distance due to better recovery), making it harder to delineate whether rehabilitation is influencing bone healing or vice versa. We also analyzed activity parameters regressed against end point bone volume for 2 mm defects, but no relationship between specific rehabilitation parameters and bone volume was revealed, likely because the 2 mm bone healing quantification lacked variability. Nonlinear algebraic models from ten independent evolutions were generated using DataModeler's model creation function. Model creation was constrained to a 10-min simulation, and only robust models were considered for analysis using the “Robust On” function. Each model received a unique fit (1 − R^2^) and complexity scores and was plotted as a function of these two terms. 108 models with complexity <100 and a 1 − R^2^ value <0.2 were selected as the “fittest” models observed at the knee of the Pareto front. The complexity score is based on the mathematical equation of the model. Low-complexity models have mathematical equations that describe a system with relatively simple terms (e.g., a small number of parameters, basic mathematical operations, and minimal interactions between variables). We wanted to minimize complexity while maximizing accuracy to investigate robust models to depict relationships relevant to biological systems. Although the exact values of the accepted complexity and accuracy scores are arbitrary, we selected these inclusion criteria to examine over 100 diverse models. These models were then analyzed using the “VariablePresence” and “VariableCombinations” functions to identify the influential rehabilitation parameters and parameter combinations. Next, the top parameter combinations were selected and used as a filter setting for the next round of model generation. Another round of nonlinear algebraic models from 10 independent evolutions was generated using only the top three rehabilitation parameters as input variables (running distance, running duration, and rest time). These models were further evaluated using the “ResponsePlotExplorer” and “SurfacePlotExplorer” functions to visualize the 2D and 3D response of bone volume as a function of running distance, as well as the combination of running distance and rest time. To avoid overfitting, the algorithm leaves the top of the 3D response plot open to indicate areas of uncertainty due to poor representation from experimental animal data. This region of uncertainty is also present in the white region of the contour plot. A Mathematica Notebook script was used to overlay red dots representing data points from each animal on the 3D surface plot and the corresponding 2D contour plot to reveal areas where the model is well represented.

### Finite element analysis

#### Segmentation and mesh generation

**FIG. 8.**
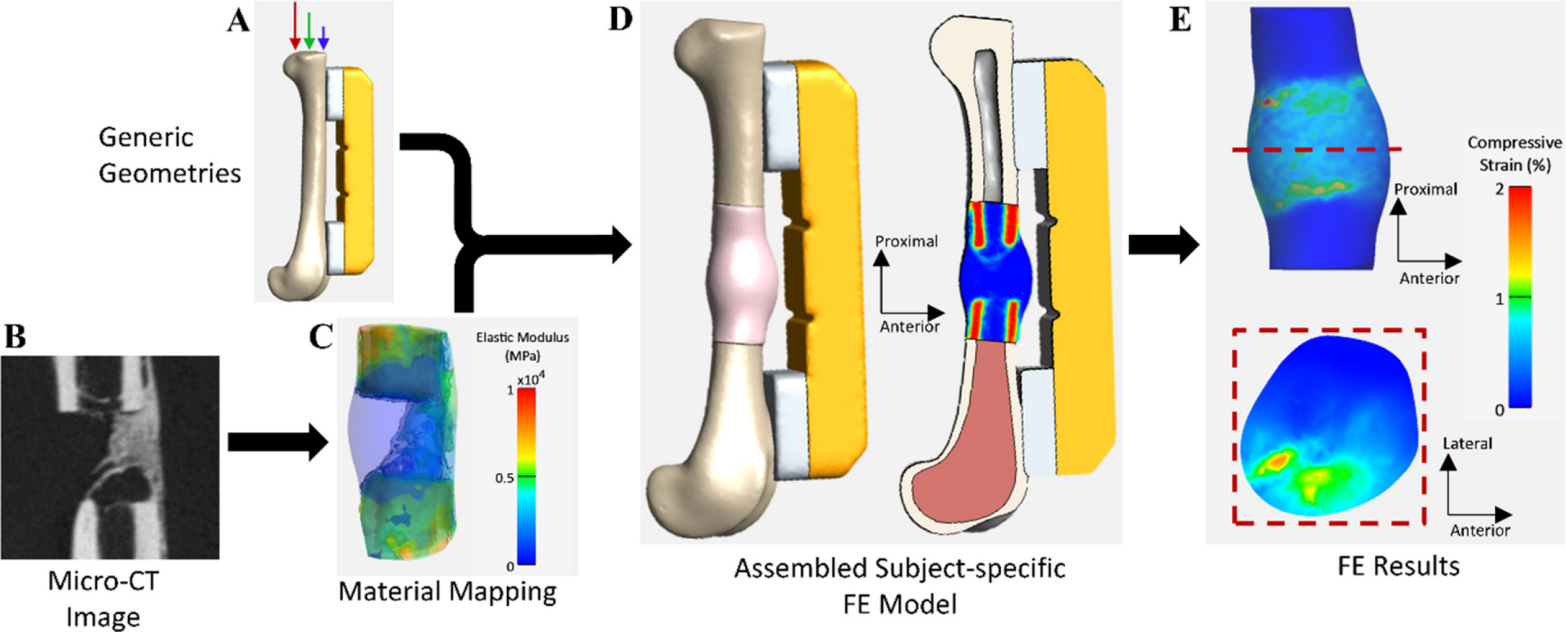
Illustration of an FE workflow employing subject-specific data to approximate local mechanics in surgically created healing bone defects. (a) Depiction of the generic geometry of the femur, risers, and fixation plate utilized in all FE models to simulate loading during rehabilitation activity. Arrows at the proximal end of the femur represent the application of spatially varying loads during rehabilitation. (b) MicroCT scans of the defect with newly mineralized tissue and parts of intact bone were captured at week 4 post-injury. (c) Position-dependent elastic modulus was assigned in the defect region on a subject-specific basis based on local microCT intensity. (d) Each assembled FE model was constructed by integrating the generic geometric models with subject-specific defect geometry. The cross-sectional view illustrates the trabecular bone and the spatial variation of elastic modulus within the defect region. (e) Representative FE results demonstrate the spatial variation of compressive strain within the defect and the influence of mineralization and bridging on strain values. Top view of the strain distribution at a cross section (red dashed line) is shown inside the dashed box.

At weeks 2 and 4 post-injury, local mechanical parameters were assessed via longitudinal FE simulations conducted on 3 mm segmental defect models from low distance control and restricted rehabilitation groups. FE models for the 2 mm defects were not created, as the healing outcomes of these defects were not significantly influenced by their rehabilitation activity. In addition, due to a lack of data for implementing subject-specific boundary conditions, the sedentary control and unrestricted groups were excluded from the FE study. As a result, the FE study consisted of thirteen 3 mm defects, where 6 of the defects achieved union after 8 weeks of recovery. First, idealized configurations of the stainless-steel risers and fixation plate used for fracture stabilization were constructed alongside left femur models, as all subjects had a 3 mm defect on their left femur [Fig. 8(a)].^5^ The femur model was divided into cortical and trabecular bone, with a hollow region representing bone marrow [Fig. 8(d)]. Defect geometries, including ectopic regions and portions of the intact femur, were delineated via semi-automated segmentation of the microCT scans acquired at weeks 2 and 4 post-injury [Fig. 8(b)] utilizing the MIMICS software (version 24.0, Materialize, Leuven, Belgium). For subjects with partial or no bridging, the outer border of the healing region was difficult to discern in the microCT image data, so we manually generated a region boundary that ensured the inclusion of volume between the femurs and enabled subject-specific material assignment within the healing region. Subsequently, FE meshes consisting of 10-node tetrahedral elements were generated for all parts using the 3-Matic software (version 16.0, Materialise, Leuven, Belgium), with the target element size for each part chosen based on previous mesh convergence studies.^5^

#### Material properties and boundary conditions

The resulting FE model was imported to FEBio Studio (Version 2.8, Salt Lake City, UT).^94^ Parts with incompatible meshes were tied together using the “tied elastic” feature.^95^ All materials were represented using an isotropic neo-Hookean hyperelastic constitutive model.^80,93,96,97^ Our preceding study empirically determined the material coefficients (elastic modulus, *E*, and Poisson's ratio, *υ*) for the stainless-steel riser and the fixation plate (*E* = 200 000 MPa, *υ* = 0.3 for risers; *E* = 525 MPa, *υ* = 0.4 for fixation plate).^5^ The elastic moduli for cortical and trabecular regions of the femur were derived from a prior study on rats of comparable age to our subjects (*E* = 8803 MPa, *υ* = 0.33 for cortical bone; *E* = 2169 MPa, *υ* = 0.33 for trabecular bone).^5,98^ We established a correlation between the intensity of the microCT images of hydroxyapatite phantoms and their density, and combined it with another equation that relates hydroxyapatite density to the elastic modulus.^98^ This allowed us to assign a position-dependent elastic modulus, *E*, within the healing defect region using the “image map” tool in FEBio Studio^99^ and the following equation:

$$E=4.49*10^{-4}\times {\left(I-19.5\right)}^{1.87},$$ (1)

where *I* is the microCT image intensity in the Hounsfield Unit (HU) and *E* is the elastic modulus in MPa [Fig. 8(c)]. This equation was applied to regions with intensities of 100 HU or higher. Regions below 100 HU were assigned a constant elastic modulus of *E* = 0.022 MPa to represent non-mineralized soft tissue. This was based on a study by Klosterhoff *et al.*,^80,93,96,97^ who found that this value resulted in the best match to experimental results for the finite simulations in their study.^5^ All FE elements with an elastic modulus exceeding 631.6 MPa were classified as mineralized, corresponding to 388 mg HA/cm^3^, which is half the density of intact cortical bone.^5^ Mineralization threshold and voxel count have an inverse relationship, so increasing the threshold beyond 388 mg HA/cm^3^ would decrease the mineralized voxel count and vice versa.

The proximal end of the femur was subjected to a spatially varying pressure load, while the distal femur was fully constrained, consistent with our prior research.^5^ Unlike our prior research, subjects in the current study did not have instrumented strain sensors on the fixation plates.^93^ We utilized strain values measured from previous studies to determine the loading needed to mimic the wheel running activity of the subjects at weeks 2 and 4. In one of our studies, *in vivo* tensile strain on the fixation plate was recorded for animals in rehabilitation conditions who achieved union after week 8 of recovery.^46^ Through a pilot study, the fixation plate strain for subjects who exhibited nonunion after 8 weeks was measured. Using an iterative process, we identified the distributed pressure load that produced tensile (first Principal Lagrange) strain on the simulated fixation plate, aligning with experimentally measured strain values (supplementary material Fig. 5). For the six union subjects, the loading conditions mirrored those from the previous study,^46^ while for the seven nonunion subjects, the boundary conditions were based on the pilot study (supplementary material Table 1).^100^ We assessed the compressive (third Principal Lagrange) and shear (Maximum Shear Lagrange) strains in elements within the segmental defects. The FEBio solver (Version 4.8) was used for all FE analyses.^101^ A nonlinear, incremental-iterative solution procedure was used based on the Broyden-Fletcher-Goldfarb-Shanno algorithm (BFGS) quasi-Newton method.

### Statistical analysis

All data were reported as mean ± standard deviation (SD). Significance was determined at p < 0.05. Mixed-effect ANOVA with Tukey's multiple comparisons test and animal as the random factor was used to analyze the impact of rehabilitation conditions and time on weekly activity parameters. The mixed-effects model accounts for shared locomotor behavior within our bilateral animal model by treating animal identity as a random factor, thereby adjusting for the non-independence of measurements from the two limbs of the same subject. Two-way ANOVA with Sidak's multiple comparisons test was used to analyze bone formation as a function of rehabilitation conditions and time for 2 and 3 mm defects independently. Bone formation within defects were analyzed as independent observations after we tested for within-animal dependence. Linear regression of end point bone volume in contralateral limbs showed no significant relationship, indicating minimal shared variance (supplementary material Fig. 6). This supports defects being analyzed as independent observations since mixed-effects modeling, with animal as a random factor, would yield results equivalent to the two-way ANOVA. All activity and bone healing statistical calculations were performed using GraphPad Prism 10 software (version 10.4.1). Data from FE analysis is represented as volume-weighted mean ± standard deviation (SD), and all statistical analyses were conducted using Origin 2024 (OriginLab, Northampton, MA).

## SUPPLEMENTARY MATERIAL

See the supplementary material for additional information relevant to the mechanical modeling, rehabilitation outcomes, and bone healing responses. They include (1) effects of rehabilitation and housing conditions on longitudinal running activity, (2) relationships between local shear strain distributions and subsequent bone volume, (3) correlations between strain in mineralized and non-mineralized tissues and defect bone volume, (4) radiographs showing defect-size-dependent healing outcomes, (5) fixation plate microstrain measurements used to inform finite element model boundary conditions, (6) application and visualization of spatially varying pressure on the proximal rat femur, and (7) regression analysis of contralateral limb bone volumes.

## Data Availability

The data that support the findings of this study are available within the article and its supplementary material.
